# Trust issues: Adolescents' epistemic vigilance towards online sources

**DOI:** 10.1111/bjdp.12559

**Published:** 2025-03-26

**Authors:** Pip Brown, Michaela Gummerum

**Affiliations:** ^1^ Department of Psychology University of Warwick Coventry UK

**Keywords:** adolescents, epistemic vigilance, online behaviour, selective trust

## Abstract

Development of epistemic vigilance towards online information is crucial for adolescents in the context of widespread online ‘information pollution’. Children have demonstrated selective mistrust of webpages with typographical but not semantic errors. We used a selective trust task to investigate whether this pattern changes through adolescence. Participants read two pairs of sources about scientific topics, each pair containing a webpage with either semantic or typographical errors. When asked novel factual questions, which source participants drew answers from indicates the degree of selective trust in the source. As anticipated, age group significantly predicted selective trust scores, with older adolescents (*N* = 222, 16–20 years, *M* = 18 years) receiving higher scores than younger adolescents (*N* = 153, 11–16 years, *M* = 13.7 years.). While this age effect was present in both typographical and semantic conditions, it was particularly pronounced for semantic errors. Additionally, pre‐exposure to an accuracy prompt was not a significant factor in selective trust scores, demonstrating some limitations in the utility of this prime for more complex selective trust decisions. We theorize that semantic errors may have more salience than typographical errors for older adolescents' selective trust decisions, whereas younger adolescents place more emphasis on a visual understanding of source credibility.

**Statement of Contribution**

- In this study we used a selective trust task to find out what kind of errors adolescents pay attention to when deciding which online sources to trust.
- In comparison to younger adolescents, older adolescents showed more selective trust in accurate over inaccurate articles.
- This was particularly the case when the inaccuracies were semantic rather than typographical.
- Pre‐exposure to an accuracy prompt was not a significant factor in selective trust behaviour.

## INTRODUCTION

Adolescents and young adults are often described as ‘Digital Natives’, highlighting their ease and adeptness with technologies they have grown up using. However, this characterization can overlook adolescents' struggles with the evaluative and critical aspects of digital literacy. Rather than simply transferring critical print‐media skills to the digital context, the novelty and variety of online sources point to the need for supporting adolescents in developing strategies towards digital textual analysis (Leu et al., 2017).

Cultivating digital literacy in adolescents is important not only for their individual epistemic health but also to empower adolescents to actively counter the spread of misinformation and conspiracy theories. According to the WHO in early 2020, internet users were exposed to an ‘infodemic’, dealing with a profusion of information generally and misinformation specifically. The COVID‐19 pandemic has demonstrated the dangers of this: Across countries, there were strong associations between endorsement of COVID misinformation and disinclination to follow government guidelines (Roozenbeek et al., 2020). Similarly, exposure to misinformation resulted in decreased intentions to receive the COVID‐19 vaccine among those who had hitherto stated their willingness (Loomba et al., 2021). Given that adolescents increasingly desire and are expected to make autonomous decisions that can have wide‐ranging consequences for themselves and others (Zimmer‐Gembeck & Collins, 2006), it is important to understand how adolescents make credibility judgements of online information.

### Do adolescents evaluate the credibility of online information effectively?

Even at the initial stage of search engine use, eye‐tracking studies indicated that adolescents ignore deeper cues to semantic relevance in favour of superficial cues, such as boldness or the size of font (Dinet et al., 2010), and engage in unstructured, unguided and fragmentary online reading strategies (Zhang, 2013). Preadolescents (10–11 years) show significant divergence between linear offline text reading and largely non‐linear online reading behaviour (Sung et al., 2015), which is associated with disorientation and cognitive overload (Conklin, 1987), and is likely to impede active and strategic critical processes underlying both the construction of meaning (Coiro, 2003) and the formation of credibility judgements (Kirschner & van Merriënboer, 2013).

When evaluating the credibility of online information, Coiro et al. (2015) found that 12‐ to 13‐year‐olds widely used irrelevant criteria to establish the credibility of online information, such as looking for a copyright symbol. Although 20% of participants did understand effective eligibility criteria (e.g., source corroboration, author expertise), most struggled to find evidence to support these criteria, and some simply did not use them. Most participants also struggled to know how to proceed if they were faced with more than one website with conflicting information.

Furthermore, there is a gap between adolescents' knowledge about credibility evaluations of online sources and their actual behaviour. While Flanagin and Metzger (2010) found that children and adolescents talked seriously about the importance of information credibility and analytical effortful and deliberate strategies for evaluating it, in their behaviour, they tended to resort to a more heuristic decision‐making process, concerned with what ‘looks right’ or ‘feels right’ (McPherson et al., 2014). Younger adolescents tended to overestimate their online critical reading abilities (Miller et al., 2012) or simply failed to interrogate the accuracy of any online information (Zhang, 2013).

Older adolescence marks a transition point, where the demands of work or university study can require more sophisticated critical digital literacy skills. As those aged 16–24 years spend more time online than any other age group (Ofcom, 2023a), it is particularly important to establish what kind of discernment decisions older adolescents and young adults make about online sources. Yet, older adolescents' digital literacy seems to present a similar picture to younger adolescents. University students' reliability judgments of online information were often not informed by information about sourcing (List et al., 2016), with college students often judging Google results to be more credible if they appear higher on the results page (Hargittai et al., 2010). Students often relied on ‘weak signals’ such as article appearance when investigating the credibility of webpages (Wineburg & Mcgrew, 2017). Asked to evaluate the trustworthiness of a satirical news article, 83 out of 125 students failed to recognize it as such, either judging it as credible or rejecting it based on irrelevant details like appearance or top‐level domain. Features like website appearance and links to credible news organizations were taken as proxies for trustworthiness (Wineburg et al., 2020). This approach leaves readers vulnerable to inappropriate use of the representativeness heuristic (Tversky & Kahneman, 1974) with the presence of easily falsifiable visual elements wrongly taken to be evidence for the actual credibility of the source.

### Selective trust in online text

Much of the research explored above could be characterized as an investigation into the epistemic vigilance of digital users. Epistemic vigilance is a suite of abilities that help humans to manage the veracity of information (Sperber et al., 2010). These might include source monitoring (i.e., making trust determinations based on the characteristics of the information source) and content evaluation (i.e., comparing the incoming information with extant store of information through metacognitive monitoring).

Selective trust paradigms (e.g., Koenig et al., 2004) are particularly useful for exploring this ability as they can test both source and content vigilance in an indirect way and are suitable for participants at varying stages of language development. In a selective trust experiment, participants are exposed to two sources of information on an unfamiliar topic. The participant is then asked a question (e.g., where do Polar bears live?) that can only be answered using information from either source (e.g., source A: South Pole; source B: North Pole). Thus, participants have to rely on the sources to answer that question. Which answer participants provide gives an indication of which source they trust.

Source monitoring capabilities seem to emerge at an early age (Sperber et al., 2010). In face‐to‐face communication, even pre‐school children use criteria like age and expertise when trying to differentiate between reliable and unreliable speakers, tracking accuracy (Harris & Corriveau, 2011), spontaneously looking for information from people who have been previously accurate (Birch et al., 2008). However, although primary‐aged children are sensitive to frequency (Pasquini et al., 2007) and severity of errors (Einav & Robinson, 2010), once independently reading, they demonstrate a bias towards trusting written information over oral testimony (Einav et al., 2018).

Much less is known about children and adolescents' selective trust in online texts. The challenge of judging the credibility of online information relates to the difficulty in finding cues to author benevolence and expertise, two key features that readers look for in source evaluation (Mascaro & Sperber, 2009). For scientific subjects, young people acquire and use knowledge about source expertise from early on in development, with secondary‐school students holding assumptions about which domain experts are more useful to listen to even without prior knowledge about the subject (Porsch & Bromme, 2010) and acting on this knowledge (Keil et al., 2008). In everyday internet use, however, the ultimate source of information might be hidden or not readily interpretable (Strømsø et al., 2013) leaving readers to extrapolate author expertise and benevolence from scant or missing information.

Given this paucity in source information, it is important to consider the impact of content on the nature of online selective trust decisions. Of particular interest in the context of misinformation are adolescents' responses to different types of errors. Einav et al. (2020) showed that 8‐ to 10‐year‐olds demonstrated epistemic vigilance towards webpages with errors, but only when these errors were typographical and not semantic. Participants were given a version of the selective trust task using three pairs of printed‐out webpages on different child‐friendly topics. In each pair, one webpage contained errors: one containing typographical errors and two containing semantic errors (exaggerations or factual inaccuracies). When asked questions that drew on different information from the accurate versus inaccurate webpages, participants used the accurate source above chance level only in the typographical condition. The same was true for the subgroup of children who correctly identified which was the inaccurate webpage for each condition. Further, only 16 out of 48 participants reported considering the overall accuracy of the text they read when they made decisions about which webpage to trust, often citing plausibility instead. Thus, in this age group, typographical errors seem to be more salient for selective trust than semantic errors.

Typographical and semantic errors have two different task demands: Monitoring for textual inaccuracy requires looking for consistency between the text and an internal orthographic standard. Semantic errors are text, that is grammatically and typographically correct, but that involve violations of prior knowledge, fact or logic (Silverstein, 1972). It is thus possible that children's difficulty with semantic errors reflects a difficulty with metacognition, particularly knowledge they have about themselves as a person and the task (i.e., metacognitive knowledge; Flavell, 1979). Indeed, studies have shown that young learners are not proficient in metacognitive self‐appraisal (e.g., ‘Do I know this?’), which affect their understanding and learning (see Paris & Winograd, 1990). If, because of this, children fail to consider overall source accuracy, they may fall back on a simpler plausibility judgement when answering questions. This tendency towards trusting background knowledge and intuitions to make plausibility judgements despite evidence of another's expertise has been seen in a similar age group to the preteens in Einav et al. (2020): In contrast to 7‐ to 8‐year‐olds who copied a known expert when trying to retrieve prizes from a puzzle box, 9‐ to 10‐year‐olds copied less and used their own flawed causal reasoning to produce a strategy (Lucas et al., 2017).

There is some evidence that older adolescents are better than younger adolescents at engaging with deeper, more systematic semantic processing when making relevance judgments in an online search setting (Dinet et al., 2010). Thus, in contrast to children, adolescents may be able to utilize better‐developed metacognitive knowledge and self‐appraisal to recognize semantic errors and use them to make trust decisions.

From a dual‐process perspective (Gilbert et al., 1990), it is more cognitively effortful to overcome the ‘default bias’ towards accepting incoming information as true. However, the fact that, when prompted, children could identify most errors across error types in Einav et al.'s (2020) study suggests that semantic errors were encoded, although this information did not appear to be used to reject the inaccurate webpage. The fact that in the semantic condition, those that identified which was the inaccurate webpage but still did not choose the accurate webpage above chance level suggests a failure to consider overall accuracy.

### Priming for vigilance

If these findings reflect a general failure to attend to the overall accuracy of sources, rather than a specific failure to encode semantic errors, then it is possible that the introduction of an accuracy prime may improve vigilance to overall errors in this task. Pennycook and Rand (2022b) emphasize the role that attention plays in sharing misinformation online. According to this perspective, misinformation sharing happens not because people do not care about accuracy; they are just distracted and need prompting to attend to accuracy. Accuracy primes can thus help people use their latent desire to share accurate information. This has been effectively demonstrated with both political (Fazio, 2020) and COVID‐19 misinformation (Pennycook et al., 2020). The most effective primes in discouraging adults' intentions to share fake news were a headline veracity judgement task paired with feedback about their choices (Pennycook & Rand, 2022a).

The effect of this prime has not yet been explored in non‐adult samples. It is not clear yet whether this prime, which has largely been investigated regarding sharing political misinformation, would prompt adolescents to pay attention to semantic inaccuracies in selective trust decisions. However, if semantic errors are not utilized in selective trust decisions because of a failure to account for overall source accuracy, then an accuracy prime should ameliorate this.

### The current research

The current study adapted Einav et al.'s (2020) task to investigate whether adolescents' selective trust behaviour towards online webpages differs when sources are typographically or semantically inaccurate. We focused on adolescents for two reasons. Compared with children, adolescents are reaching epistemic autonomy from parents and teachers, become more socially and politically engaged and aware, and are expected and desire to make autonomous decisions with sometimes wide‐ranging consequences based on available information (Zimmer‐Gembeck & Collins, 2006). Second, with online and social media becoming the favoured source of information for adolescents (Ofcom, 2023b), it is important to understand whether and how people in this age group critically evaluate the accuracy of online sources.

Participants read pairs of webpages on scientific topics, one accurate and one inaccurate (containing typographical or semantic errors). When asked factual questions concerning the content of the webpages, participants could draw answers from either webpage, thus giving a measure of selective trust and epistemic vigilance towards malinformation. If younger children's lack of selective mistrust in semantic errors reflects under‐developed metacognitive knowledge and self‐appraisal, then it is possible that adolescents who may have more developed metacognitive skills (Schneider, 2008) could use these semantic error cues more readily when deciding which sources to trust. Therefore, older adolescent participants should show epistemic vigilance in both the semantic and the typographical condition (Hypothesis 1). We expected that those who could spot the errors when prompted would be more likely to demonstrate selective trust and that this would be the case across age groups (Hypothesis 2).

Additionally, we investigated whether an accuracy prime affects selective mistrust in the inaccurate webpages. We predicted that those primed would demonstrate greater epistemic vigilance (i.e., selective mistrust of inaccurate websites) than non‐primed participants (Hypothesis 3a). Alternatively, if selective trust is based on a more rudimentary problem of metacognition, where although semantic errors are recognized, this nevertheless does not motivate trust decisions and we would not expect a priming effect (Hypothesis 3b). As previous research has mainly employed accuracy primes for sharing online (mis‐) information, we predicted that adolescents (like adults in previous research) would be less likely to share inaccurate information when exposed to an accuracy prime (Hypothesis 4).

## METHOD

### Participants

A G* Power analysis (Faul et al., 2007) was undertaken using a conservative effect size of *r* = .30 drawn from the selective trust literature (e.g., Einav et al., 2020; Eyden et al., 2013) which indicated a total *N* of 352 for a mixed design with two between‐subject factors (early/mid‐ and older adolescents/prime vs. non‐prime) and one within‐subject factor (error type) for sufficient power at .95 with an alpha of .05.

Adolescents were recruited from the undergraduate student body at the University of Warwick, from local sixth forms/post‐16 institutions, and secondary‐school institutions in central England. In compliance with our institutional ethical procedures, we were required to give participants a choice as to whether they revealed their age. A total of 37 declined to give their age in years. While we did not know the exact age of these participants, we know where they were recruited from. Given this and the nature of the differing demands regarding young people's ability to search and critically evaluate new information and pedagogical approaches of the UK key stage school system (National Literacy Trust, 2018), we chose to group participants by age group rather than using age as a continuous variable. This yielded a group of early/mid‐adolescents in the secondary stage of education (11–16 years) and older adolescents (16–20 years) in the tertiary stage of education.

Of the older adolescents recruited, 222 (*M*_age_ = 18.03 years, *SD* = 0.81) participants yielded complete data and met the requirements of fluency in English and independence in reading as measured by self‐report. Early to mid‐adolescents (11–16 years) were recruited from two local secondary schools. Of those recruited, 153 (*M*_age_ = 13.74 years, *SD* = 1.45) yielded complete data and met the inclusion criteria. For a more detailed account of inclusion/exclusion practices, see Supporting Information. Incomplete data or responses of participants not meeting the inclusion/exclusion criteria were deleted.

### Design

This study employed a 2 (Prime condition: prime v. no‐prime) × 2 (Error Condition: Typographical v. semantic) experimental design. Prime condition served as a between‐subject variable and error condition as a within‐subject variable. Age group (early/mid v. older adolescents) served as another independent variable.

We collected three main dependent variables: Selective trust, intentions of sharing the presented websites and errors spotted (on incorrect websites). For exploratory analyses, we also assessed participants' motivations for their website choice.

## MATERIALS AND MEASURES

### Prime

**FIGURE 1:**
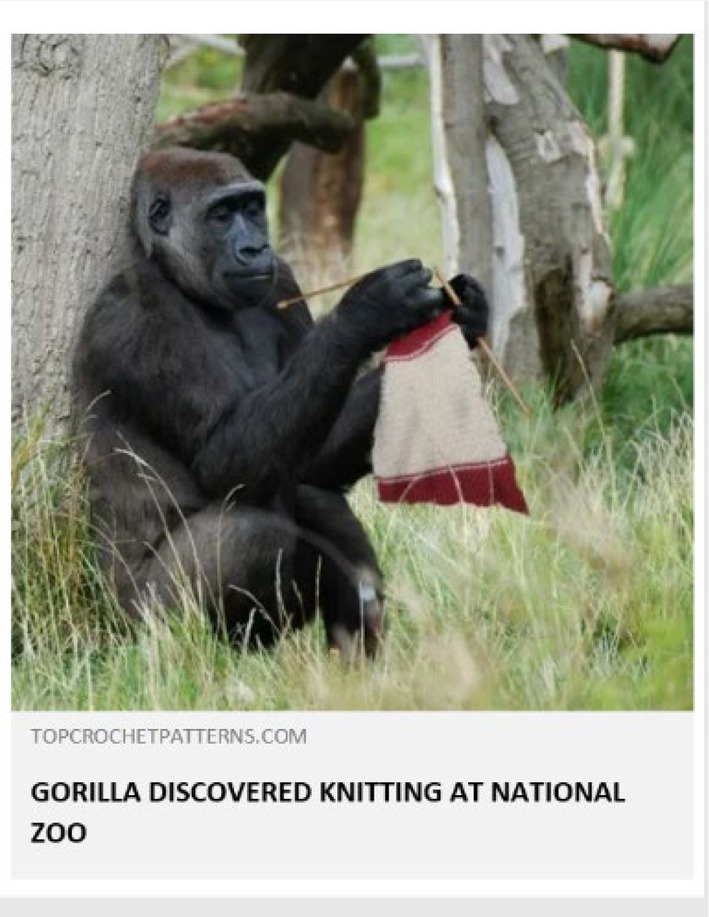
Example of a fake news headline item in the prime task.

Following procedures established by Pennycook et al. (2020), five ‘Fake news headlines’ were collected and modified from existing examples or fabricated completely by the researcher. The remaining five real news headlines were taken from science news stories appearing in a variety of mainstream online news organizations. All headlines were in the style of a social media news link, containing a headline, web address and picture (see example in Figure 1).

### Selective trust task

For each error condition (typographical, semantic), we created one accurate and one inaccurate webpage about octopus intelligence and antibiotic resistance, constructed in the Wix website editor utilizing their free templates. For the purposes of greater verisimilitude, each differed in content, illustrations, and had minor changes to the font and layout and general presentation to resemble real websites more closely. Because of this compromise between ecological validity and control, it was crucial that participants were asked directly about their motivations for choice. This allows greater context for what would otherwise be a binary forced‐choice decision. The text used was adapted from Wikipedia Simple English (https://simple.wikipedia.org) and the Encyclopaedia Britannica Online (https://www.britannica.com), shortened, simplified and supplemented with misinformation where appropriate.

All websites were pilot tested. In Pilot 1, twenty 18‐ to 19‐year‐olds and twenty six 16‐ to 17‐year‐olds were asked to identify errors using Qualtrics's Heatmap function. In order to avoid floor effects for our early to mid‐adolescent age group, in Pilot 2, fifty four 11‐ to 16‐year‐olds conducted the same tasks on similar but age‐appropriate stimuli with slightly simplified language and modifications made to some of the errors. Screenshots of the websites used can be found in Figure S1. Each inaccurate webpage contained four introduced errors. Thus, for each errors condition (typographical v. semantic), there were four errors. Table S1 gives an overview of the errors.

### Knowledge test (selective trust)

After each pair of websites was presented, participants were asked to answer four knowledge questions using only the information from the websites (eight knowledge questions altogether). They were reminded that they could scroll up to read each webpage again and that the webpages might say different things. The full list of questions as well as the conflicting answers provided by the accurate and inaccurate websites can be found in Table S2. For each of the four questions after each pair, participants were awarded 1 point if the answer given had been drawn from the accurate webpage and 0 points if it had been taken from the inaccurate webpage. Participants who gave inappropriate or incomplete answers, or who drew answers from neither webpage, were excluded from analysis. The higher the score, the more participants demonstrated selective trust in the accurate webpages.

### Sharing intentions

After each webpage, participants were asked to imagine that they and their friends were doing a project on these topics and were asked to indicate how likely they were to share the webpages with their friends on a 5‐point Likert‐like scale from extremely likely (score of 1) to extremely unlikely (score of 5).

### Identification of errors

Participants were told that ‘You might have noticed that some of these webpages contained errors’ and to click on the errors if they could spot them (for the inaccurate webpages). Receptive areas were constructed using the Heatmap function on Qualtrics to match the coordinates of participants' clicks against the actual location of the errors. Participants were awarded 1 point for a click within the receptive fields of the errors, for a maximum total of 4 points per incorrect website. Multiple clicks within the same field counted only once.

### Motivation for website choice

Participants were asked an open‐ended question as to what factors they considered when they were choosing which webpage/information to use for the knowledge tests.

### Procedure

The study received full ethical approval from Warwick University. Participants completed the study online on Qualtrics. Only participants (and parents/guardians where participants were under 18) who provided informed consent were able to take part.

Participants were randomly assigned to one of the two Prime conditions using the Qualtrics randomization function. In the prime group (*n* = 178), they were shown a series of ten real and fake news headlines (randomly presented) and asked to indicate whether the headlines were accurate or inaccurate. After each judgment, participants were informed about the headline accuracy. The no‐prime group (*n* = 197) proceeded straight to the main experiment.

Afterwards, all participants engaged in the selective trust task. They were shown two pairs of websites about science and health topics, one pair of websites for the typographical and semantic error conditions, respectively. Within each pair, one website contained either typographic or semantic errors, and one website contained no errors. The presentation order of error conditions was randomized for each participant using the Qualtrics randomization function. Similarly, within each error condition, websites with and without errors were presented randomly. After each of the four websites, participants were asked to indicate their sharing intentions. After each pair of websites, participants were asked to answer four knowledge questions using only the information from the two websites.

Participants were then asked to identify errors on the inaccurate websites. Each inaccurate webpage was shown again, with errors highlighted and explained, and the correct information provided. Participants were finally asked an open‐ended question about their motivations for their website choice.

Participants were debriefed and informed that we had fabricated the webpages and the inaccurate headlines in the prime task. Participants were presented with information from the FaktaBaari organization aiming to help people identify and deal with online misinformation.

## RESULTS

### Selective trust

**FIGURE 2:**
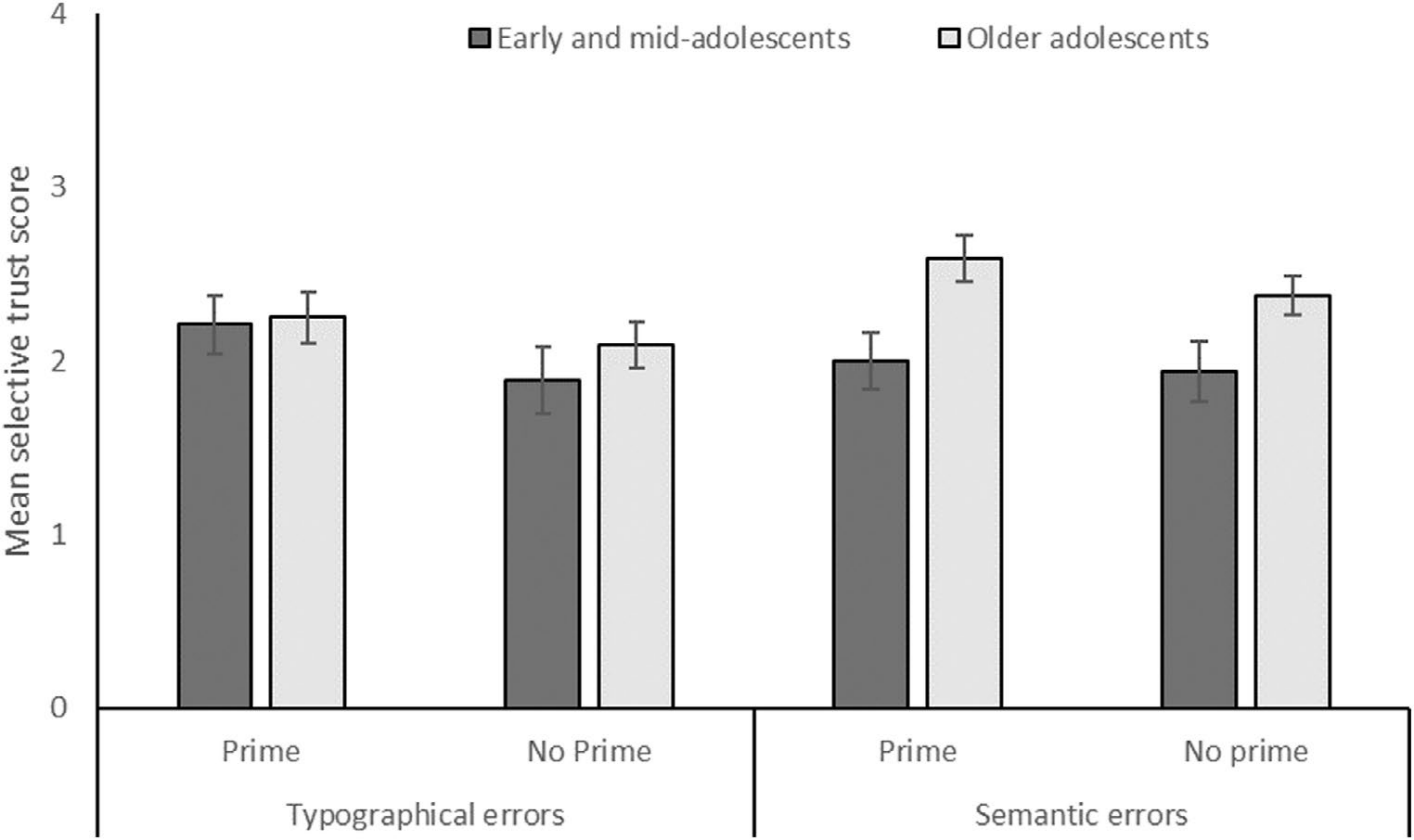
Mean selective trust scores by age group, error type and prime conditions. Error bars display standard errors.

Figure 2 shows the mean selective trust scores (i.e., mean number of knowledge questions answered correctly) by age group, error type (typographical, semantic) and prime condition. A log‐link Generalized Estimating Equations (GEE) model with a Poisson distribution was fitted to the selective trust data with the predicted main effects of Prime (prime/non‐prime), Error Condition (typographical/semantic), Age Group (early/mid‐adolescents v. older adolescents) and the predicted interactions of Error × Age Group and Prime × Age Group. Age Group significantly predicted selective trust, *W*(1) = 6.66, *p* = .01, *β* = −.23, *SE* = .08, CI [−0.39, −0.07], with older participants receiving higher scores than younger ones. This main effect was qualified by an interaction of Error Condition × Age Group, *W*(1) = 3.90, *p* = .048, *β* = .18, *SE* = .09, CI [0.001, 0.36], as predicted by Hypothesis 1. Older adolescents received higher selective trust scores than early/mid‐adolescents, particularly for semantic errors, but there was no age difference in selective trust for typographical errors (Figure 2).

In contrast to Hypothesis 3a but in line with Hypothesis 3b, neither the main effect of Prime, *W*(1) = 2.42, *p* = .12, *β* = −.08, *SE* = .06, CI [−0.20, 0.03], nor the Prime × Age Group interaction, *W*(1) = 0.12, *p* = .90, *β* = −.01, *SE* = .11, CI [−0.24, 0.21], reached statistical significance.

In line with the selective trust literature, we conducted Wilcoxon signed‐rank tests with Bonferroni‐corrected alpha levels (*α* = .006) to examine whether selective trust significantly differed from chance (a score of 2). Older adolescents who had not been primed showed significantly higher selective trust scores in the semantic error condition (*M* = 2.36, *SD* = 1.21) than would be expected by chance, *z* = 3.26, *df* = 126, *p* = .003. Similarly, older adolescents who had been primed showed significantly higher selective trust in the semantic condition (*M* = 2.58, *SD* = 1.22) than would have been expected by chance, *z* = 1.77, *df* = 95, *p* < .001. None of the other selective trust scores differed significantly from chance.

In addition, we investigated age differences in selective trust for each of the four knowledge questions in the typographical and semantic condition, respectively, thereby treating selective trust as a forced‐choice response. These analyses and results are reported in the Supporting Information.

### Error spotting

We then investigated whether participants could identify errors on the inaccurate website when prompted. As displayed in Table 1, in most conditions, participants identified at least one error, with the exception of the early/mid‐adolescents in the semantic error conditions. Across conditions, on average, less than half of the errors were spotted.

**TABLE 1:** Percentage and mean (and *SD*) errors spotted by age group and error condition.

Age group	Error condition	Prime	*N*	Percent zero errors spotted (%)	Mean (*SD*) errors spotted
Early/mid‐adolescents	Typographical	Prime	83	24.1	2.22 (1.59)
		No prime	68	23.5	2.13 (1.60)
	Semantic	Prime	82	45.1	1.15 (1.32)
		No prime	67	59.7	0.81 (1.15)
Older adolescents	Typographical	Prime	92	38.0	1.61 (1.53)
		No prime	125	35.2	1.74 (1.53)
	Semantic	Prime	92	30.4	1.27 (1.18)
		No prime	125	41.6	1.16 (1.25)

*Note*: There are 2 missing responses in the early/mid‐adolescents age group. This is due to those participants exercising their right to not respond to a question.

Testing Hypothesis 2, a GEE with a Poisson distribution with the predicted main effects of Age Group, Errors Spotted, Error Condition and the interactions between Error Condition × Errors Spotted and Age Group × Error Condition × Errors Spotted revealed the previously reported main effect of Age Group on selective trust, *W*(1) = 21.91, *p* < .001, *β* = −.41, *SE* = .09, CI [−0.58, −0.24] and a significant main effect of Errors Spotted, *W*(1) = 65.87, *p* < .001, *β* = .10, *SE* = .02, CI [0.05, 0.14]: The more errors participants spotted on the inaccurate websites, the higher their selective trust. These main effects were qualified by a significant interaction of Age Group × Error Condition × Errors Spotted, *W*(2) = 19.23, *p* < .001, *β* = .15, *SE* = .03, CI [0.08, 0.21]. Follow‐up non‐parametric correlations indicated that for early/mid‐adolescents selective trust correlated significantly and positively with errors spotted in both the typographical, *τ*(140) = .37, *p* < .001, and semantic, *τ*(135) = .30, *p* < .001 conditions. For older adolescents, selective trust only significantly and positively correlated with errors spotted in the semantic, *τ*(215) = .25, *p* < .001, but not the typographical condition, *τ*(215) = .07, *p* = .21.

### Sharing intentions

Testing Hypothesis 4, a GEE model with a linear‐link function tested the main effects of Prime (No prime, Prime), Age Group, Error Condition as well as the interactions of Prime × Age Group and Prime × Error Condition on intentions to share inaccurate websites. A significant Prime × Age Group effect emerged, *W*(1) = 12.83, *p* < .001, *β* = −.76, *SE* = .22 CI [−1.20, −0.35]. Early/mid‐adolescents who were primed were less likely to share websites (*M* = 3.24, *SD* = 1.23) than those who were not primed (*M* = 2.98, *SD* = 1.22). This priming effect was reversed among older adolescents (primed: *M* = 2.88, *SD* = 1.33; not primed: *M* = 3.39, *SD* = 1.32).

Following comments during the review process, we also investigated whether sharing intentions differed for accurate websites. These exploratory analyses are reported in the Supporting Information.

### Motivations

We conducted exploratory analyses on participants' answers regarding their website choice. While participants' answers were varied and sometimes inconsistent with their actual behaviour in the selective trust task, they helped contextualize the selective trust results.

**FIGURE 3:**
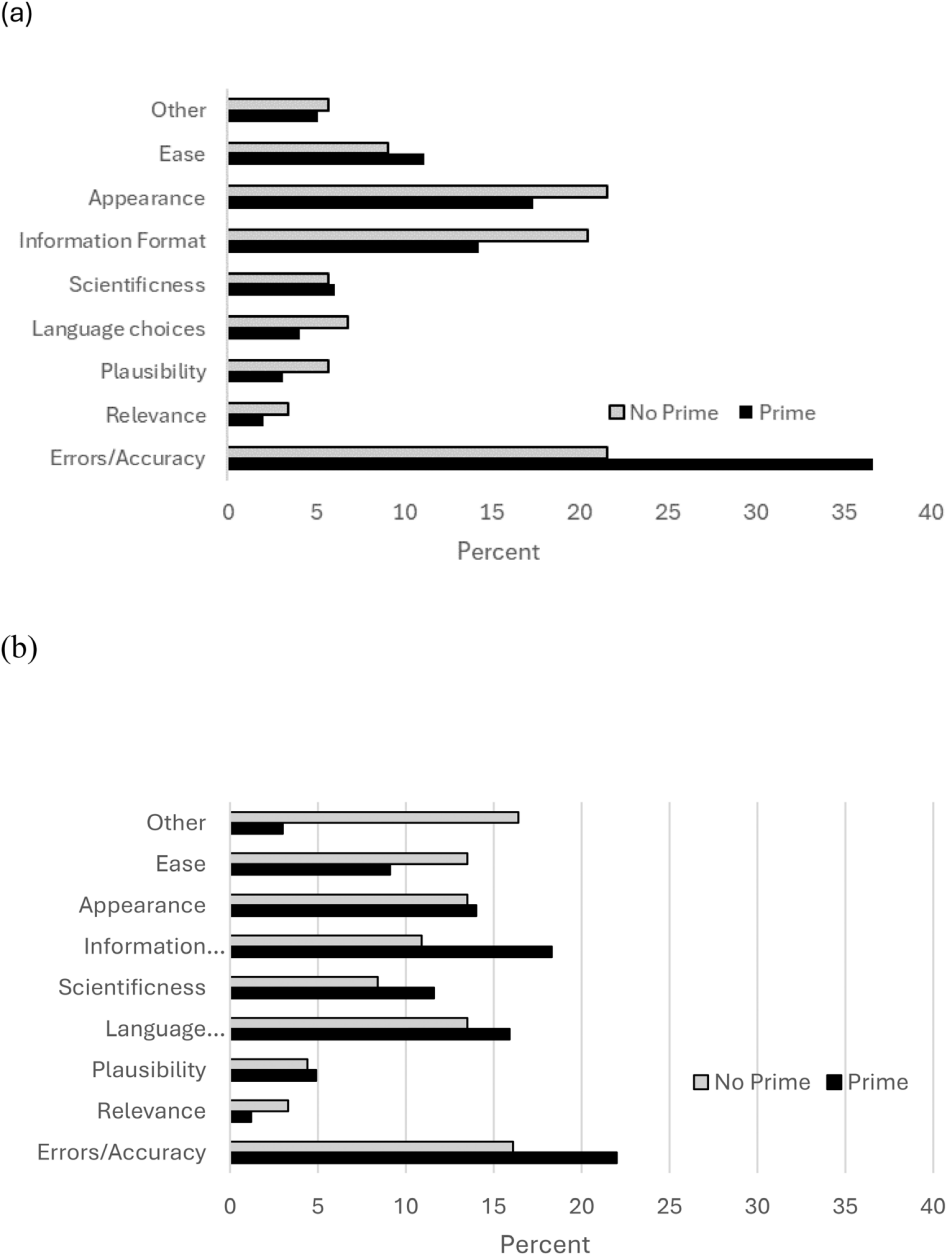
Percentage categories used in responses to open‐ended question for website choice by prime for (a) early/mid‐adolescents and (b) older adolescents.

A preliminary content analysis was performed, with an independent coder, unfamiliar with the stimuli in question, coding the responses into initial categories using Nvivo. These codes were then combined into broader categories (see Figure 3). Please note that the content of participants' answers could be coded into multiple categories. Most strikingly, across age groups and condition more than 60% of participants failed to mention information accuracy in their answers (Figure 3). Considerations of appearance (responses mentioning aesthetics, colour, readability and detail) accounted for most of participant responses. However, the possibility of experimenter demand cannot be discounted here; these responses were elicited after participants spotted errors. Some answers, therefore, may constitute a post hoc rationalization of what may have been a choice of convenience (such as which website was nearest the answer box). At least one participant cited website characteristics that did not exist (such as whether they recognized the webpage that had been created by the experimenter). Another participant recognized that they were making a choice between an accurate and an inaccurate webpage and decided to choose answers from the inaccurate webpage. To explore the relation between participant's stated website choice motivations and their selective trust behaviour, Generalized Estimating Equations (GEE) model with a Poisson distribution was fitted to the selective trust data. This showed a significant main effect of accuracy mentions, *W*(1) = 55.80, *p* < .001, *β* = −.30, *SE* = 0.06, CI [−0.42, −0.19], with those that did not mention accuracy in their motivations showing a lower selective trust score than those who did mention accuracy. A significant interaction of Accuracy Mention × Age Group, *W*(1) = 12.19, *p* = .002, *β* = −.28, *SE* = 0.08, *p* = .02, CI [−0.44, −0.11] indicated that this was more pronounced for older than younger participants (see Table 2 for descriptives).

**TABLE 2:** Selective trust when accuracy was mentioned as motivation for website choice.

	Frequency of accuracy mentioned	Mean (*SD*) selective trust score when accuracy was mentioned	Mean (*SD*) selective trust score when accuracy was not mentioned
Early/mid‐adolescents			
Prime	31/86	2.56 (1.35)	1.44 (1.35)
No prime	18/75	2.65 (1.40)	1.49 (1.34)
Older adolescents			
Prime	33/82	3.00 (1.25)	2.15 (1.34)
No prime	41/112	2.56 (1.41)	1.96 (1.25)

*Note*: There are 6 missing responses in the early/mid‐adolescents age group. This is due to those participants exercising their right to not respond to a question.

We also explored whether the accuracy prime affected whether participants were more likely to mention accuracy in their motivations. Participants in the prime condition (41.8%) were more likely than expected to mention accuracy, whereas those in the no‐prime condition (34.7%) were less likely than expected to mention accuracy, but this difference was not significant, *χ* ^2^(1) = 3.57, *p* = .059.

## DISCUSSION

The present study predicted a developmental improvement across adolescence in selective trust behaviour towards inaccurate online sources, with an additional improvement after exposure to an accuracy prime.

### Selective trust

As expected, the ability to demonstrate selective trust improved across adolescence, with older adolescents performing better than early/mid‐adolescents, despite the slightly simplified stimuli used by the younger group. This developmental difference in selective trust in favour of older adolescents was more pronounced for semantic than typographical errors. Across age groups, those that were more likely to spot errors when prompted showed higher levels of selective trust. This effect of error spotting on selective trust was shown for both types of errors in early/mid‐adolescents, but only for semantic errors in older adolescents.

The findings provide an interesting extension to Einav et al. (2020) and indicate potential developmental patterns of selective trust and epistemic vigilance in online sources across adolescence. These developmental patterns should be replicated in future, ideally longitudinal, research. Eight‐ to 10‐year‐olds in Einav et al. (2020) demonstrated epistemic vigilance for typographical but not semantic errors. Early/mid‐adolescents in the current study showed similar levels of selective trust in the typographical and semantic conditions, and both types of errors (when spotted) were diagnostic for their selective trust decisions. Conversely, semantic errors seemed to be more salient and diagnostic than typographical errors for older adolescents. This may reflect a growing sophistication in adolescents' approach to judging source trustworthiness, in the sense that they may no longer be satisfied with a shallower reading of whether it ‘looks right’ and instead are engaging in a higher level of metacognitive processing, checking factual content against their store of background knowledge (Dinet et al., 2010).

Selective trust was significantly related to number of errors spotted. However, even when explicitly prompted to find errors on the inaccurate websites, participants' ability to spot these errors was relatively low compared with previous research by Einav et al. (2020). One reason for this discrepancy might be that in Einav et al.'s (2020) study, children were presented with a print‐out of the websites used in the experiment and thus were scrutinizing a paper version. The current study was conducted entirely online. Previous research (see meta‐analysis by Delgado et al., 2018) showed that reading comprehension is higher in paper‐based than online text. In a study with middle‐school students, Fisher et al. (2011) found that readers were particularly prone to miss important details in online compared with paper‐based texts (see also Singer & Alexander, 2017). To accommodate longer text passages, our participants were required to scroll down to read the webpages, with each page read and compared vertically rather than laterally, which has been shown to negatively affect recall (Wästlund, 2007). Furthermore, typographical errors may have been overlooked as in social media settings, non‐standard spelling or punctuation, such as ‘expressive lengthening’ or adding superfluous repeated letters to words, is used to convey tonal information (Fersini et al., 2016). Overall, this research highlights the ease with which even rather blatant errors and inaccurate information are overlooked by adolescents in online contexts and how this might affect their decisions based on this information.

### Priming accuracy

We introduced an empirically tested (Pennycook et al., 2020) accuracy prime (vs. no prime) to assess whether failure to selectively mistrust erroneous sources could be explained by participants inattention to accuracy generally, rather than not encoding semantic or typographical errors. The accuracy prime did not affect selective trust decisions in either age group or error type. However, older adolescents, who received the accuracy prime, were less likely to share inaccurate and more likely to share accurate websites.

There are several potential reasons for the failure of our accuracy prime to affect selective trust. First, it is most likely that our longer more complex selective trust task was simply too different from Pennycook et al.'s (2020) Twitter/X sharing decisions. Nevertheless, this explanation runs counter to theories which primarily account for misinformation sharing/acceptance in terms of cognitive reflection and shallow processing (Pennycook & Rand, 2022a, 2022b). If susceptibility to misinformation is influenced mainly by the attentional demands of the internet, then accuracy prompts should still be as affective for encouraging selective trust as they are for encouraging sharing discernment.

Second, notwithstanding failures to replicate (Roozenbeek et al., 2021), political partisanship may mediate the effectiveness of accuracy prompts (Martel et al., 2023 *preprint*; Osmundsen et al., 2021). We decided to use apolitical stimuli because of the risk of politically and socially salient information showing a continued influence after the study. It is, however, possible that non‐political misinformation may not register as egregious or important enough for accuracy primes to make a measurable behavioural difference. This might be particularly important for informing developmental misinformation interventions, in that adolescents' online information ecosystems may not divide neatly into partisan lines. We did not measure partisanship in our participants, and it is not clear how far the particularly polarized US political landscape would map readily onto a UK developmental sample.

In keeping with the approach of many ‘classical’ priming studies in different areas of psychology (e.g., syntactic priming, Ledoux et al., 2007; see Mahowald et al., 2016, for a meta‐analysis) as well Pennycook et al.'s (2020) original study using the accuracy prime, we did not introduce an independent manipulation check. However, we believe the fact that participants who had been primed (versus those not primed) were slightly more likely to mention accuracy in their motivations can serve as a sanity check. While research (e.g., Hauser et al., 2018) cautions against ‘default’ manipulation checks which could affect responses independent of the prime, future research might explore ways in which the success of this prime can be assessed validly in adolescents.

### Motivations

Within the motivations for choice data, although errors and accuracy were mentioned frequently, another main motivation concerned visual aspects of the webpages, such as the visual layout, the colour of the webpage, ease of reading, pictures and references to ‘scientific’ or ‘professional’ appearances. This aligns with the literature describing shallow, primarily superficial judgements based on appearance‐based heuristics. However, it is also possible that accuracy concerns play more of a primary role in decision‐making, but the difficulties in remembering past decision‐making processes result in the most memorable/salient factors (such as colour) being recalled first (i.e., representativeness, Tversky & Kahneman, 1974).

As participants answered the motivation question after having been prompted to search for errors, it may be that the error‐spotting task is affecting their motivation responses. For early/mid‐adolescents, the metacognitive and memory demands of reflecting on their motivations might result in a response that is less reflective of their actual motivations during the task. This could explain why stated accuracy motivations were less predictive of selective trust behaviour in younger participants. The motivation analysis was exploratory; thus, in order to better understand these ambiguities, it will be necessary to probe participants in future work. More qualitative and in‐depth methods might be required to achieve this, for example, dyadic‐collaborative methods. Indeed, much of the adolescent online information environment has an explicitly social aspect to it (e.g., sharing, liking, commenting, etc.), and collaboration on a task appears to produce more sophisticated scientific arguments in younger adolescents (e.g., Teasley, 1995). Arguing for one's opinion also makes visible the decision‐making process (Gummerum et al., 2008) and might provide richer context and detail to the selective trust paradigm.

### Limitations and future research

Future research should address other limitations of the current research. As discussed, when designing the website, we had to make a compromise between experimental control and creating websites that are realistic for participants while avoiding floor and ceiling effects. Future research could make the websites more generic across conditions, which might, however, come at a cost of external validity.

Our results suggest that epistemic vigilance towards particularly semantic errors might be driven by developing metacognitive abilities, particularly metacognitive knowledge and self‐appraisal (Paris & Winograd, 1990). While research on (the development of) other aspects of metacognition, particularly metacognitive regulation (e.g., identification and selection of appropriate learning strategies), abounds (see Schneider, 2008), studies on the development of metacognitive self‐appraisal (‘Do I know this?’) is comparatively scarce and focus on self‐appraisal in reading comprehension. Interestingly, many of these studies (e.g., Winograd & Johnston, 1982) use a similar error‐spotting paradigm as the one employed in the current study to assess metacognitive knowledge. Nevertheless, future studies should attempt to assess metacognitive knowledge independently, for example, using self‐report measures capturing participants feelings‐of‐knowing or using non‐verbal methods (e.g., text lookbacks, emotional responses; see Lai, 2011; Winograd & Johnston, 1982).

We suggested that the low error‐spotting rate in the current study was due to adolescents' more shallow reading of online compared with print text. This hypothesis could be tested in future research, for example, by directly comparing adolescents' selective trust and error spotting in the same text presented in print or online. In order to improve adolescents' comprehension of online texts, we might apply and test strategies used by professional fact‐checkers, who practice lateral reading, locating the source within an interconnected network of information and using strategies, such as ‘critical ignoring’ of (irrelevant) sources (Kozyreva et al., 2023).

### CONCLUSIONS

This paper demonstrated developmental differences in epistemic vigilance towards online sources in adolescence. These results support the notion that older adolescents are better able to demonstrate selective mistrust towards inaccurate sources, particularly semantically inaccurate ones. Yet, our data also highlight that adolescents may well require additional and targeted support in developing epistemic vigilance skills towards online information sources specifically. Given that adolescents are increasingly required to make decisions with sometimes far‐reaching consequences in social, career and consumer contexts, researchers and educators should continue to find ways to enable adolescents to make good‐quality decisions based on good‐quality (online) information.

## AUTHOR CONTRIBUTIONS

**Pip Brown:** Conceptualization; writing – original draft; writing – review and editing; formal analysis; investigation; methodology; data curation; project administration. **Michaela Gummerum:** Supervision; writing – review and editing; project administration; conceptualization; methodology; formal analysis.

## CONFLICT OF INTEREST

The authors declare no conflicts of interest.

## Supporting information


Data S1.

## Data Availability

Data available upon request to corresponding author.

## References

[bjdp12559-bib-0001] Birch, S. A. J. , Vauthier, S. A. , & Bloom, P. (2008). Three‐ and four‐year‐olds spontaneously use others' past performance to guide their learning. Cognition, 107(3), 1018–1034. 10.1016/j.cognition.2007.12.008 18295193

[bjdp12559-bib-0002] Coiro, J. (2003). Reading comprehension on the internet: Expanding our understanding of reading comprehension to encompass new literacies. The Reading Teacher, 56(5), 458–464. http://starchild.gsfc.nasa.gov/

[bjdp12559-bib-0003] Coiro, J. , Coscarelli, C. , Maykel, C. , & Forzani, E. (2015). Investigating criteria that seventh graders use to evaluate the quality of online information. Journal of Adolescent and Adult Literacy, 59(3), 287–297. 10.1002/jaal.448

[bjdp12559-bib-0004] Conklin, J. (1987). Hypertext: An introduction and survey. Computer, 20, 17–41.

[bjdp12559-bib-0005] Delgado, P. , Vargas, C. , Ackerman, R. , & Salmerón, L. (2018). Don't throw away your printed books: A meta‐analysis on the effects of reading media on reading comprehension. Educational Research Review, 25, 23–38. 10.1016/j.edurev.2018.09.003

[bjdp12559-bib-0006] Dinet, J. , Bastien, J. M. C. , & Kitajima, M. (2010). What, where and how are young people looking for in a search engine results page? Impact of typographical cues and prior domain knowledge. Conference Internationale Francophone Sur I'Interaction Homme‐Machine, 262.

[bjdp12559-bib-0007] Einav, S. , Levey, A. , Patel, P. , & Westwood, A. (2020). Epistemic vigilance online: Textual inaccuracy and children's selective trust in webpages. British Journal of Developmental Psychology, 38(4), 566–579. 10.1111/bjdp.12335 32342990

[bjdp12559-bib-0008] Einav, S. , & Robinson, E. J. (2010). Children's sensitivity to error magnitude when evaluating informants. Cognitive Development, 25(3), 218–232.

[bjdp12559-bib-0009] Einav, S. , Rydland, V. , Grøver, V. , Robinson, E. J. , & Harris, P. L. (2018). Children's trust in print: What is the impact of late exposure to reading instruction? Infant and Child Development, 27(6). 10.1002/icd.2102

[bjdp12559-bib-0010] Eyden, J. , Robinson, E. J. , Einav, S. , & Jaswal, V. K. (2013). The power of print: Children's trust in unexpected printed suggestions. Journal of Experimental Child Psychology, 116(3), 593–608. 23981273 10.1016/j.jecp.2013.06.012

[bjdp12559-bib-0011] Faul, F. , Erdfelder, E. , Lang, A. G. , & Buchner, A. (2007). G* power 3: A flexible statistical power analysis program for the social, behavioral, and biomedical sciences. Behavior Research Methods, 39(2), 175–191. 17695343 10.3758/bf03193146

[bjdp12559-bib-0012] Fazio, L. K. (2020). Pausing to consider why a headline is true or false can help reduce the sharing of false news. Harvard Kennedy School Misinformation Review, 1(2). 10.37016/mr-2020-009

[bjdp12559-bib-0013] Fersini, E. , Messina, E. , & Pozzi, F. A. (2016). Expressive signals in social media languages to improve polarity detection. Information Processing and Management, 52(1), 20–35. 10.1016/j.ipm.2015.04.004

[bjdp12559-bib-0014] Fisher, D. , Lapp, D. , & Wood, K. (2011). Reading for details in online and printed text: A prerequisite for deep reading. Middle School Journal, 42, 58–63.

[bjdp12559-bib-0015] Flanagin, A. J. , & Metzger, M. J. (2010). Kids and credibility: An empirical examination of youth, digital media use, and information credibility. MIT Press. www.macfound.org

[bjdp12559-bib-0016] Flavell, J. H. (1979). Metacognition and cognitive monitoring: A new area of cognitive developmental inquiry. American Psychologist, 34, 906–911. 10.1037/0003-066X.34.10.906

[bjdp12559-bib-0017] Gilbert, D. T. , Krull, D. S. , & Malone, P. S. (1990). Unbelieving the unbelievable: Some problems in the rejection of false information. Journal of Personality and Social Psychology, 59(4), 601–613.

[bjdp12559-bib-0018] Gummerum, M. , Keller, M. , Takezawa, M. , & Mata, J. (2008). To give or not to give: Children's and adolescents' sharing and moral negotiations in economic decision situations. Child Development, 79(3), 562–576. 18489413 10.1111/j.1467-8624.2008.01143.x

[bjdp12559-bib-0019] Hargittai, F. E. , Fullerton, L. , Menchen‐Trevino, E. , & Yates Thomas, K. (2010). Trust online: Young adults' evaluation of web content. International Journal of Communication, 4, 468–494. http://ijoc.org

[bjdp12559-bib-0020] Harris, P. L. , & Corriveau, K. H. (2011). Young children's selective trust in informants. Philosophical Transactions of the Royal Society, B: Biological Sciences, 366(1567), 1179–1187. 10.1098/rstb.2010.0321 PMC3049091 21357240

[bjdp12559-bib-0021] Hauser, D. J. , Ellsworth, P. C. , & Gonzalez, R. (2018). Are manipulation checks necessary? Frontiers in Psychology, 9, 998. 29977213 10.3389/fpsyg.2018.00998 PMC6022204

[bjdp12559-bib-0022] Keil, F. C. , Stein, C. , Webb, L. , Billings, V. D. , & Rozenblit, L. (2008). Discerning the division of cognitive labor: An emerging understanding of how knowledge is clustered in other minds. Cognitive Science, 32(2), 259–300. 10.1080/03640210701863339 19759842 PMC2744112

[bjdp12559-bib-0023] Kirschner, P. A. , & van Merriënboer, J. J. G. (2013). Do learners really know best? Urban legends in education. Educational Psychologist, 48(3), 169–183. 10.1080/00461520.2013.804395

[bjdp12559-bib-0024] Koenig, M. A. , Clément, F. , & Harris, P. L. (2004). Trust in testimony: Children's use of true and false statements. Psychological Science, 15(10), 694–698. 15447641 10.1111/j.0956-7976.2004.00742.x

[bjdp12559-bib-0025] Kozyreva, A. , Wineburg, S. , Lewandowsky, S. , & Hertwig, R. (2023). Critical ignoring as a core competence for digital citizens. Current Directions in Psychological Science, 32(1), 81–88. 10.1177/09637214221121570 37994317 PMC7615324

[bjdp12559-bib-0026] Lai, E. R. (2011). Metacognition: A literature review. Always Learning: Pearson Research Report, 24, 1–40.

[bjdp12559-bib-0027] Ledoux, K. , Traxler, M. J. , & Swaab, T. Y. (2007). Syntactic priming in comprehension: Evidence from event‐related potentials. Psychological Science, 18(2), 135–143. 17425534 10.1111/j.1467-9280.2007.01863.x PMC1852484

[bjdp12559-bib-0028] Leu, D. J. , Kinzer, C. K. , Coiro, J. , Castek, J. , & Henry, L. A. (2017). New literacies: A dual‐level theory of the changing nature of literacy, instruction, and assessment. Journal of Education, 197(2), 1–18. 10.1177/002205741719700202

[bjdp12559-bib-0029] List, A. , Grossnickle, E. M. , & Alexander, P. A. (2016). Undergraduate Students' justifications for source selection in a digital academic context. Journal of Educational Computing Research, 54(1), 22–61. 10.1177/0735633115606659

[bjdp12559-bib-0031] Loomba, S. , de Figueiredo, A. , Piatek, S. J. , de Graaf, K. , & Larson, H. J. (2021). Measuring the impact of COVID‐19 vaccine misinformation on vaccination intent in the UK and USA. Nature Human Behaviour, 5(3), 337–348. 10.1038/s41562-021-01056-1 33547453

[bjdp12559-bib-0032] Lucas, A. J. , Burdett, E. R. R. , Burgess, V. , Wood, L. A. , McGuigan, N. , Harris, P. L. , & Whiten, A. (2017). The development of selective copying: Children's learning from an expert versus their mother. Child Development, 88(6), 2026–2042. 10.1111/cdev.12711 28032639

[bjdp12559-bib-0033] Mahowald, K. , James, A. , Futrell, R. , & Gibson, E. (2016). A meta‐analysis of syntactic priming in language production. Journal of Memory and Language, 91, 5–27.

[bjdp12559-bib-0034] Martel, C. , Rathje, S. , Clark, C. J. , Pennycook, G. , Van Bavel, J. J. , Rand, D. , & van der Linden, S. (2023). On the efficacy of accuracy prompts across partisan lines: An adversarial collaboration. Psychological Science, 35(4), 435–450. 10.1177/09567976241232905 38506937

[bjdp12559-bib-0035] Mascaro, O. , & Sperber, D. (2009). The moral, epistemic, and mindreading components of children's vigilance towards deception. Cognition, 112(3), 367–380. 10.1016/j.cognition.2009.05.012 19540473

[bjdp12559-bib-0036] McPherson, A. C. , Gofine, M. L. , & Stinson, J. (2014). Seeing is believing a mixed methods study exploring the quality and perceived trustworthiness of online information about chronic conditions aimed at children and young people. Health Communication, 29(5), 473–482. 24099647 10.1080/10410236.2013.768325

[bjdp12559-bib-0037] Miller, C. , Bartlett, J. , & Programme, E. (2012). Journal of information literacy “digital fluency”: Towards young people's critical use of the internet. The Journal of Information Literacy, 6(2). http://ojs.lboro.ac.uk/ojs/index.php/JIL/article/view/

[bjdp12559-bib-0063] National Literacy Trust . (2018). Fake news and critical literacy. https://nlt.cdn.ngo/media/documents/Fake_news_and_critical_literacy_‐_final_report.pdf

[bjdp12559-bib-0061] Ofcom . (2023a). Adults media literacy tracker. https://www.ofcom.org.uk/siteassets/resources/documents/research‐and‐data/data/statistics/2024/adults‐media‐literacy‐tracker/adults‐media‐literacy‐tracker‐2023‐technical‐report?v=330751

[bjdp12559-bib-0062] Ofcom . (2023b). Children's media use and attitudes. https://www.ofcom.org.uk/siteassets/resources/documents/research‐and‐data/media‐literacy‐research/children/childrens‐media‐use‐and‐attitudes‐2023/childrens‐media‐use‐and‐attitudes‐report‐2023.pdf?v=329412

[bjdp12559-bib-0038] Osmundsen, M. , Bor, A. , Bjerregaard Vahlstrup, P. , Bechmann, A. , & Petersen, M. B. (2021). Partisan polarization is the primary psychological motivation behind political fake news sharing on twitter. American Political Science Review, 115(3), 999–1015.

[bjdp12559-bib-0039] Paris, S. G. , & Winograd, P. (1990). Promoting metacognition and motivation of exceptional children. Remedial and Special Education, 11, 7–15.

[bjdp12559-bib-0040] Pasquini, E. S. , Corriveau, K. H. , Koenig, M. , & Harris, P. L. (2007). Preschoolers monitor the relative accuracy of informants. Developmental Psychology, 43(5), 1216–1226. 17723046 10.1037/0012-1649.43.5.1216

[bjdp12559-bib-0041] Pennycook, G. , McPhetres, J. , Zhang, Y. , Lu, J. G. , & Rand, D. G. (2020). Fighting COVID‐19 misinformation on social media: Experimental evidence for a scalable accuracy‐nudge intervention. Psychological Science, 31(7), 770–780. 10.1177/0956797620939054 32603243 PMC7366427

[bjdp12559-bib-0042] Pennycook, G. , & Rand, D. G. (2022a). Accuracy prompts are a replicable and generalizable approach for reducing the spread of misinformation. Nature Communications, 13, 2333. https://www.nature.com/articles/s41467‐022‐30073‐5 10.1038/s41467-022-30073-5 PMC9051116 35484277

[bjdp12559-bib-0043] Pennycook, G. , & Rand, D. G. (2022b). Nudging social media toward accuracy. The Annals of the American Academy of Political and Social Science, 700(1), 152–164. 10.1177/00027162221092342 35558818 PMC9082967

[bjdp12559-bib-0044] Porsch, T. , & Bromme, R. (2010). Which science disciplines are pertinent? ‐Impact of epistemological beliefs on students' choices. In K. Gomez , L. Lyons , & J. Radinsky (Eds.), Learning in the Disciplines: Proceedings of the 9th International Conference of the Learning Sciences (ICLS 2010)–Volume 1, Full Papers (pp. 636–642). International Society of the Learning Sciences.

[bjdp12559-bib-0045] Roozenbeek, J. , Freeman, A. L. J. , & van der Linden, S. (2021). How accurate are accuracy‐nudge interventions? A preregistered direct replication of Pennycook et al. (2020). Psychological Science, 32(7), 1169–1178. 10.1177/09567976211024535 34114521 PMC8641132

[bjdp12559-bib-0046] Roozenbeek, J. , Schneider, C. R. , Dryhurst, S. , Kerr, J. , Freeman, A. L. J. , Recchia, G. , van der Bles, A. M. , & van de Linden, S. (2020). Susceptibility to misinformation about COVID‐19 around the world. Royal Society Open Science, 7, 201199. 10.1098/rsos.201199 33204475 PMC7657933

[bjdp12559-bib-0047] Schneider, W. (2008). The development of metacognitive knowledge in children and adolescents: Major trends and implications for education. Mind, Brain, and Education, 2, 114–121. 10.1111/j.1751-228X.2008.00041.x

[bjdp12559-bib-0048] Silverstein, M. (1972). Linguistic theory: Syntax, semantics, pragmatics. Annual Review of Anthropology, 1, 349–382.

[bjdp12559-bib-0049] Singer, L. M. , & Alexander, P. A. (2017). Reading across mediums: Effects of reading digital and print texts on comprehension and calibration. Journal of Experimental Education, 85(1), 155–172. 10.1080/00220973.2016.1143794

[bjdp12559-bib-0050] Sperber, D. , Clément, F. , Heintz, C. , Mascaro, O. , Mercier, H. , Origgi, G. , & Wilson, D. (2010). Epistemic vigilance. Mind & Language, 25, 359–393.

[bjdp12559-bib-0051] Strømsø, H. I. , Bråten, I. , Britt, M. A. , & Ferguson, L. E. (2013). Spontaneous sourcing among students reading multiple documents. Cognition and Instruction, 31(2), 176–203. 10.1080/07370008.2013.769994

[bjdp12559-bib-0052] Sung, Y. T. , Wu, M. D. , Chen, C. K. , & Chang, K. E. (2015). Examining the online reading behavior and performance of fifth‐graders: Evidence from eye‐movement data. Frontiers in Psychology, 6, 665. 10.3389/fpsyg.2015.00665 26074837 PMC4446912

[bjdp12559-bib-0053] Teasley, S. D. (1995). The role of talk in children's peer collaborations. Developmental Psychology, 31(2), 207–220.

[bjdp12559-bib-0054] Tversky, A. , & Kahneman, D. (1974). Judgment under uncertainty: Heuristics and biases. Science, 185(4157), 1124–1131. 10.1126/science.185.4157.1124 17835457

[bjdp12559-bib-0055] Wästlund, E. (2007). Experimental studies of human‐computer interaction: Working memory and mental workload in complex cognition. Department of Psychology, Gothenberg University.

[bjdp12559-bib-0056] Wineburg, S. , Breakstone, J. , & Ziv, N. (2020). Educating for misunderstanding: How approaches to teaching digital literacy make students susceptible to scammers, rogues, bad actors and hate mongers (Working Paper No. A‐21322). Stanford History Education Group. Retrieved March, 2, 2021, from https://purl.stanford.edu/mf412bt5333

[bjdp12559-bib-0057] Wineburg, S. , & Mcgrew, S. (2017). Lateral reading: Reading less and learning more when evaluating digital information. Teachers College Record, 121(11), 1–40.

[bjdp12559-bib-0058] Winograd, P. , & Johnston, P. (1982). Comprehension monitoring and the error detection paradigm. Journal of Reading Behavior, 1, 61–76. 10.1080/10862968209547435

[bjdp12559-bib-0059] Zhang, M. (2013). Supporting middle school students' online reading of scientific resources: Moving beyond cursory, fragmented, and opportunistic reading. Journal of Computer Assisted Learning, 29(2), 138–152. 10.1111/j.1365-2729.2012.00478.x

[bjdp12559-bib-0060] Zimmer‐Gembeck, M. J. , & Collins, W. A. (2006). Autonomy development during adolescence. In Blackwell handbook of adolescence (pp. 174–204). Wiley.

