# Supplementary material for: Trust issues: Adolescents' epistemic vigilance towards online sources
Source: Br J Dev Psychol. 2025 Mar 26;43(3):578–94. doi: 10.1111/bjdp.12559 (PMC12351225; doi:10.1111/bjdp.12559)
Supplement: Supplementary file 1 — Data S1. [file BJDP-43-578-s001.docx]

**Supplementary Materials**

**Data Inclusion Criteria**

In the early-mid-adolescent age group, many participants had missing data, possibly due to the study's length. It was decided that for the selective trust task, if no attempt had been made to answer questions in both conditions, or if in both conditions less than two questions had been sensibly addressed, then participant data would be removed. This was the case for 79 early mid -adolescents, and 34 older adolescents. 9 older adolescent participants indicated they did not meet the language fluency requirements, and 23 across both age groups were removed due to a coding error which meant they did not complete the appropriate conditions.

**Figure S1**

*Screenshots of the Four Websites Used: (a) Typographical Condition, Accurate Webpage; (b) Typographical Condition, Inaccurate webpage; (c) Semantic Condition, Accurate Webpage; (d) Semantic condition, Inaccurate Webpage*

*(a)*


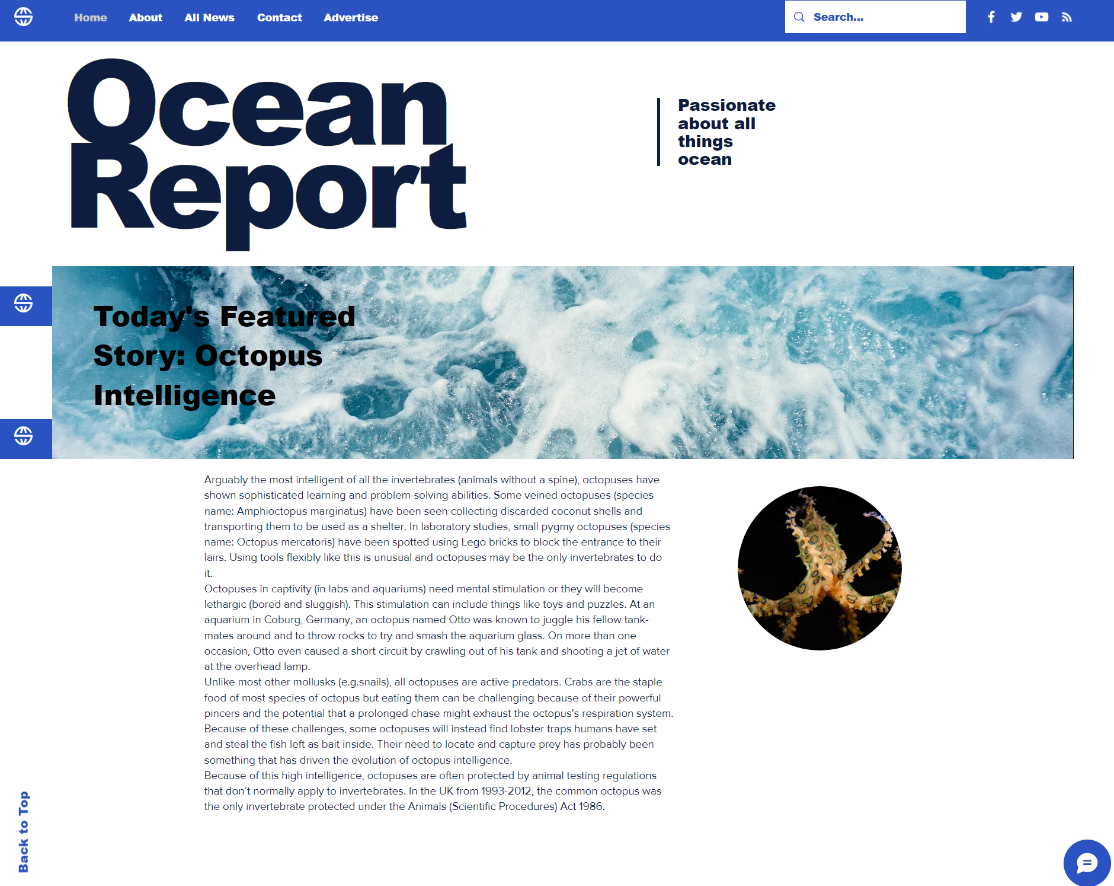


*(b)*


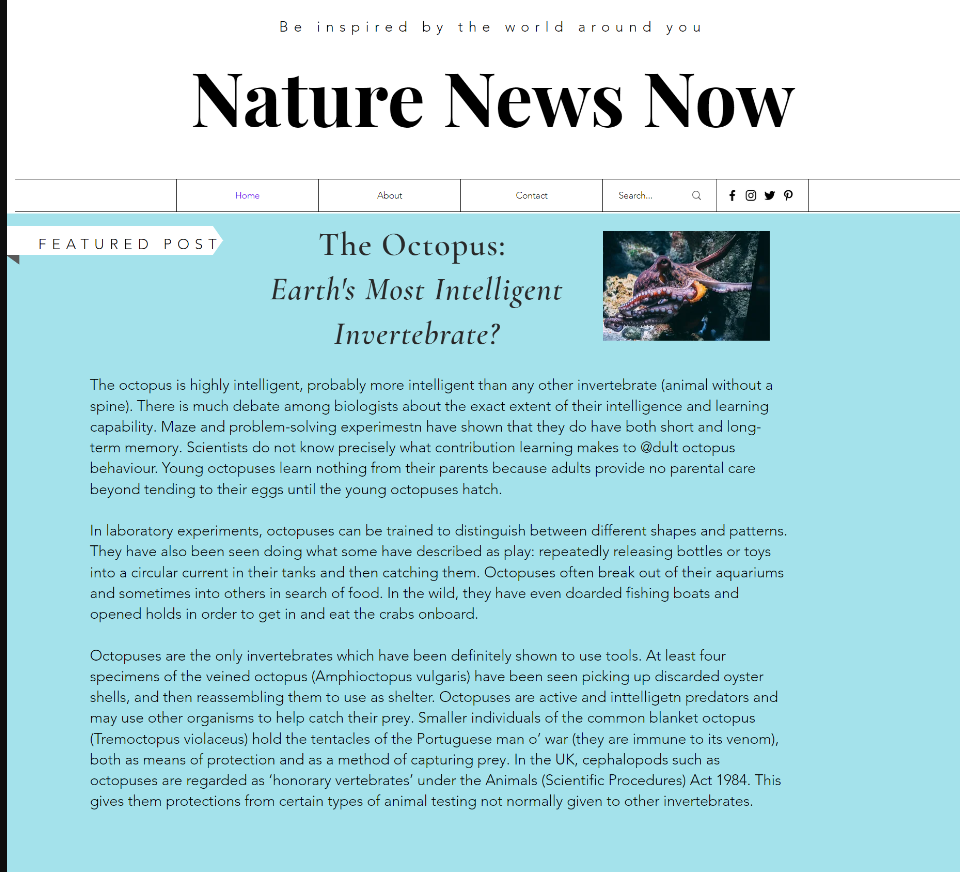


*(c)*


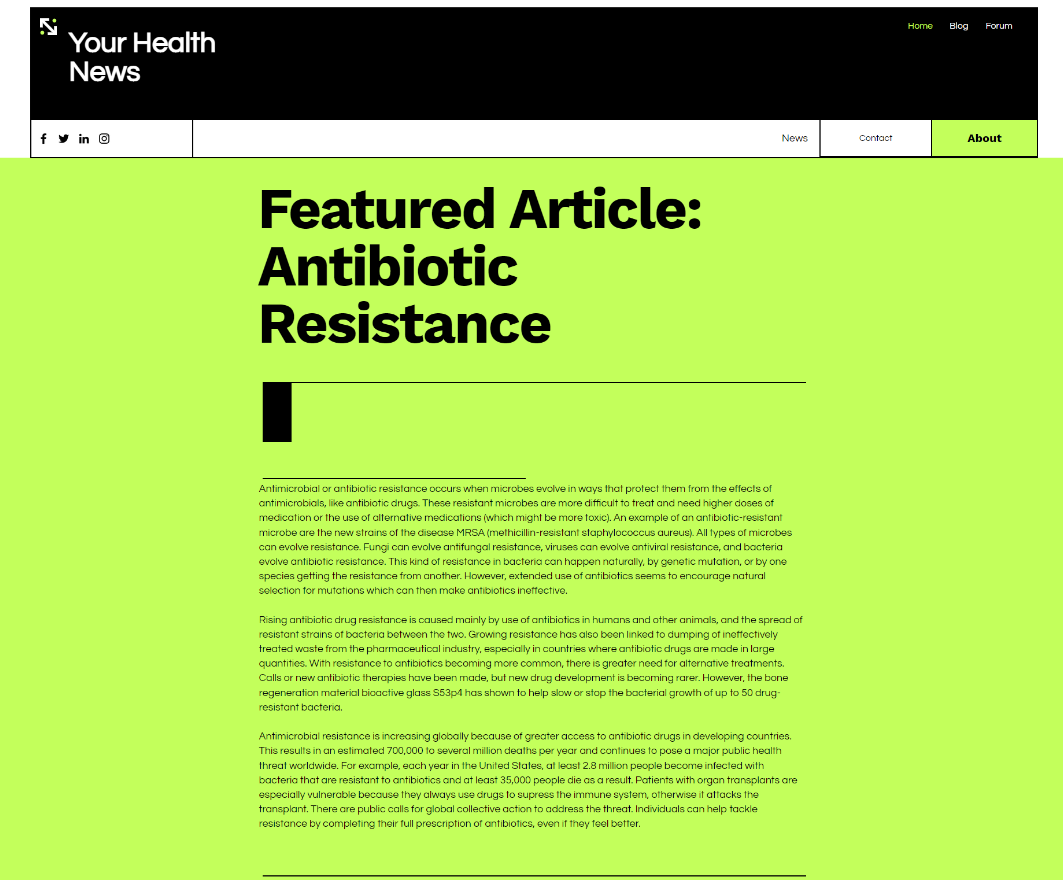


*(d)*


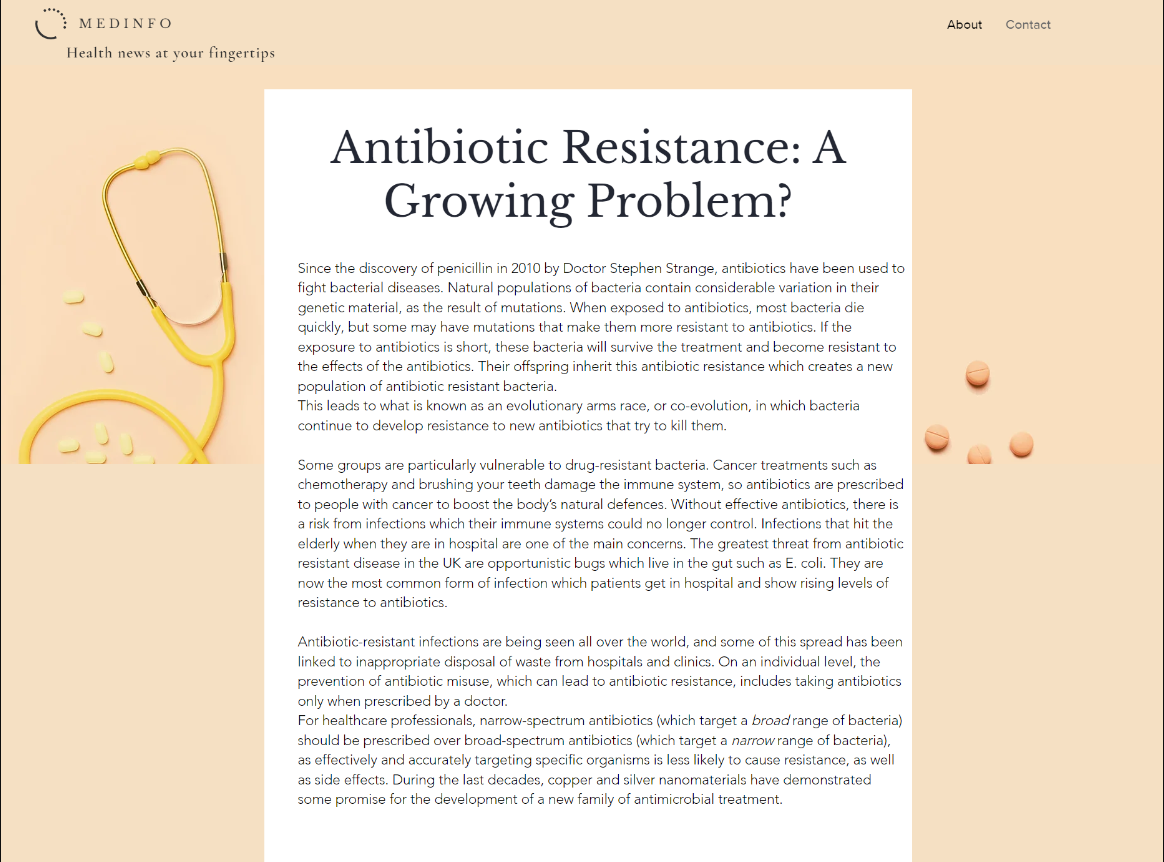


**Table S1**

*Overview of Typographical and Semantic Errors on the Incorrect Websites*

|  | Errors | | | |
| --- | --- | --- | --- | --- |
| Error condition | 1 | 2 | 3 | 4 |
| Simplified Materials | | | | |
| Typographical | experimestn | @dult | doarded | inttelligetn |
| Semantic | Penicillin discovered in 2010 | Penicillin discovered by Dr Strange | Brushing your teeth is a treatment for cancer | Definitions swapped for broad and narrow spectrum antibiotics |
| Harder Materials | | | | |
| Typographical | experimetns | too | boreded | inttelligent |
| Semantic | Penicillin discovered in 1528 | Penicillin discovered by Albert Einstein | Psychotherapy is a treatment for cancer | Definitions swapped for broad and narrow spectrum antibiotics |

*Note: In the study, the simplified materials were subsequently used for the early-mid adolescents and the harder materials for the older adolescents.*

**Table S2**

*Knowledge Questions Asked and Responses Provided by Accurate and Inaccurate Webpages*

| Question | Accurate webpage answer | Inaccurate webpage answer |
| --- | --- | --- |
| What type of shells have octopuses been seen using for shelter? | Coconut shells | Oyster shells |
| What is the Latin (Scientific) name for the Veined Octopus. You will find this in brackets after the words ‘veined octopus’ | Amphioctopus Marginatus | Amphioctopus Vulgaris |
| Apart from the veined octopus, please name ONE other species of octopus | Small pygmy octopus/octupus mercatoris | Tremoctopus Violaceus/common blanket octopus |
| What year was the Animals (Scientific Procedures) Act? | 1986 | 1984 |
| Name ONE antibiotic resistant disease | MRSA | E.coli |
| Name ONE NEW treatment for antibiotic resistant disease | Bioactive glass | Copper and silver nanomaterials |
| Name ONE way that INDIVIDUALS can help tackle drug-resistance | Take their full prescription of antibiotics even if they feel better | Take antibiotics only when prescribed by a doctor |
| APART from the elderly, name ONE group that is vulnerable to getting antibiotic resistant diseases | Organ transplant patients | Cancer patients |

**Pilot Studies: Spotting Errors on Inaccurate Websites**

As shown in Table S3, although mean error spotting scores are significantly lower for the harder than the easier stimuli in the typographical condition (N = 100, df(1), U = 4.90, Mdn = 2, *p* = .045) and the Semantic condition (N= 100, df (1), *U*= 19.63, Mdn = 1, *p* = <.001), the same task in the study itself yielded significant difference in scores in the semantic condition only (N = 381 , df(1), *U* = 5.50, Mdn = 1, *p* = .03) not the typographical (N= 380, df (1), *U* = 0.10, Mdn =2, *p*= .83) and in the opposite direction.

**Table S3**

*Descriptives: Mean Error Spotting in the Pilot Studies for Easier and Harder Materials*

| Stimuli | Condition | N | *M* | *SD* |
| --- | --- | --- | --- | --- |
| Simplified | Typographical | 54 | 2.26 | 1.54 |
|  | Semantic | 54 | 2.07 | 1.40 |
| Harder | Typographical | 46 | 1.52 | 1.50 |
|  | Semantic | 46 | 0.67 | 1.10 |

**Skewness and Kurtosis of Main Dependent Variables**

**Selective Trust**

For the typographical condition, skewness of the selective trust score was z = -0.63, for the semantic condition z = -1.93. While these are acceptable values, Kolmogorov-Smirnov tests indicated that the distribution of selective trust in both the typographical and the semantic conditions differed significantly from a normal distribution (both *p*s < .001). Kurtosis was z = -5.64 for the typographical condition and z = -3.94 for the semantic condition. Shapiro-Wilks tests indicated that the distributions differed significantly from a normal distribution (both *p*s < .001).

**Errors Spotted**

Skewness of errors spotted in the typographical conditions was z = 0.35, in the semantic condition z = 6.50. Kolmogorov-Smirnov tests demonstrated that the distribution of errors spotted in both the typographical and the semantic conditions differed significantly from a normal distribution (both *p*s < .001).

Kurtosis for errors spotted was z = -6.10 for the typographical and z = -1.78 for the semantic condition. Shapiro-Wilks tests indicated that the distributions differed significantly from a normal distribution (both *p*s < .001).

**Selective Trust as a Forced-Choice Response**

We investigated for each of the four knowledge questions in the typographical and semantic condition, respectively, whether there were age differences in whether participants used information from the accurate (v. inaccurate) website. Table S4 shows the descriptive statistics and performed chi-square tests. There were no significant age effects in the percentage of the correct website being chosen to answer the questions in the typographical error condition. However, for the semantic error condition, for two questions, older adolescents were more likely than early/mid-adolescents to pick information from the accurate website.

**Table S4**

*Percentage Choice Information from Accurate Website by Error Condition, Question, and Age Group and Associated Chi-Square Tests.*

| Error Condition | Question | Percentage Accurate Website Chosen | | *χ^2^, df, p* |
| --- | --- | --- | --- | --- |
|  |  | Early/Mid-adolescents | Older adolescents |  |
| Typographical | 1 | 50.7 | 50.2 | 0.46, 1, .50 |
|  | 2 | 55.3 | 52.7 | 0.23, 1, .63 |
|  | 3 | 44.1 | 53.0 | 2.75, 1, .10 |
|  | 4 | 64.7 | 56.4 | 2.43, 1, .12 |
| Semantic | 1 | 57.9 | 70.6 | 6.63, 1, .01 |
|  | 2 | 24.8 | 58.4 | 7.60, 1, 01 |
|  | 3 | 46.2 | 54.1 | 2.10, 1, .15 |
|  | 4 | 55.3 | 52.7 | 0.23, 1, .63 |

**Sharing Intentions of Accurate and Inaccurate Websites**

Table S5 shows how likely participants were to share inaccurate and accurate websites by Age Group, Error Type (typographical, semantic), and Prime condition. In these exploratory analyses, we included all main and higher-order effects. A repeated-measures Analysis of Variance revealed the main within-subject effect of Accuracy, *F*(1, 708) = 103.13, *p* < .001, *η^2^* = .13 as well as the significant between-subject main effects of Age Group, *F*(1, 708) = 9.32, *p* = .002, *η^2^* = .01. Overall, participants were more likely to share the accurate than the inaccurate websites, and early/mid-adolescents were less likely to share than older ones. These main effects were qualified by significant interaction effects of Accuracy x Age Group, *F*(1, 708) = 12.22, *p* < .001, *η^2^* = .02, and Accuracy x Prime x Age Group, *F*(1, 708) = 15.72, *p* < .001, *η^2^* = .02. Whereas there was no age difference in sharing intentions for the inaccurate website, early/mid-adolescents were more likely to share accurate websites than older adolescents. When primed, older adolescents became more likely to share accurate and less likely to share inaccurate websites than when they were not primed. Early/mid-adolescents were more likely to share inaccurate and less likely to share accurate websites when primed than when not primed. None of the main and interactions effects involving Error Condition reached statistical significance.

**Table S5**

*Mean (and SD) Sharing Intentions By Age Group, Error Condition, and Prime Condition, and* *Webpage Type (Accurate, Inaccurate). Higher Sharing Scores Indicate Lower Intentions to Share.*

| Age Group | Error condition | Prime | Webpage type (accurate or inaccurate) | N | Mean (SD) Sharing intentions |
| --- | --- | --- | --- | --- | --- |
| Early/Mid-adolescents | Typographical | Prime | Accurate | 81 | 2.65 (1.13) |
|  |  | Prime | Inaccurate | 81 | 3.25 (1.14) |
|  |  | No prime | Accurate | 67 | 2.51 (1.17) |
|  |  | No prime | Inaccurate | 68 | 3.09 (1.28) |
|  | Semantic | Prime | Accurate | 82 | 2.54 (1.06) |
|  |  | Prime | Inaccurate | 79 | 2.95 (1.32) |
|  |  | No prime | Accurate | 64 | 2.95 (0.98) |
|  |  | No prime | Inaccurate | 66 | 2.86 (1.15) |
| Older adolescents | Typographical | Prime | Accurate | 91 | 2.37 (1.10) |
|  |  | Prime | Inaccurate | 95 | 2.95 (1.27) |
|  |  | No prime | Accurate | 124 | 2.14 (1.06) |
|  |  | No prime | Inaccurate | 126 | 3.42 (1.24) |
|  | Semantic | Prime | Accurate | 90 | 2.27 (1.14) |
|  |  | Prime | Inaccurate | 94 | 2.81 (1.39) |
|  |  | No prime | Accurate | 124 | 2.19 (0.98) |
|  |  | No prime | Inaccurate | 125 | 3.35 (1.39 |
